# Citrus Genotype Modulates Rhizosphere Microbiome Structure and Function Under Drought Stress

**DOI:** 10.3390/plants15010077

**Published:** 2025-12-26

**Authors:** Yanqi Teng, Can Yin, Fuyin Xu, Jiyu Chen, Qiong Wu, Mingyan Ye, Yiding Liu, Kai Zhu

**Affiliations:** 1 School of Agriculture and Forestry Science and Technology, Chongqing Three Gorges Vocational College, Chongqing 404100, China; 2021170684@cqsxzy.edu.cn (Y.T.); xfy0907@126.com (F.X.); 2012170163@cqsxzy.edu.cn (J.C.); solod123@163.com (Q.W.); cqyydye@163.com (M.Y.); 2 College of Agronomy and Biotechnology, China Agricultural University, Beijing 100083, China; yincan@cau.edu.cn; 3 College of Horticulture and Landscape Architecture, Southwest University, Chongqing 400715, China; 4 Guangxi Academy of Agricultural Sciences Sugarcane Research Institute, Nanning 530007, China

**Keywords:** citrus, rhizosphere microorganisms, drought, physiological characteristics, soil indicators

## Abstract

Drought stress substantially impairs citrus growth and alters the rhizosphere microbial composition; however, the role of these microbial communities in plant drought tolerance remains poorly understood. This study investigated the rhizosphere microbial structure, soil enzymatic activities, and physicochemical properties of drought-tolerant (DR) and drought-sensitive (DS) citrus varieties under drought stress conditions. High-throughput sequencing revealed that drought significantly altered microbial community composition, reducing the bacterial Shannon diversity by about 15% and enriching Gram-negative, stress-tolerant, and potentially pathogenic bacteria, as well as plant pathogenic fungi (upregulated 25.4% in DS), while reducing undefined saprotrophs (downregulated from 76.2 to 54.0% in DS). Notably, the DR variety exhibited a more stable and complex bacterial network, with 23.5% more edges and a higher proportion of positive correlations (54.3%), higher enrichment of beneficial fungi like *Penicillium* and *Trichoderma*, and unique recruitment of mycorrhizal fungi (up to 10.2%), which were nearly absent in DS. Furthermore, soil catalase and urease activities decreased under drought stress conditions. In contrast, acid phosphatase activity increased by up to 40% in DR. Correlation analyses indicated that these microbial shifts were closely associated with changes in soil nutrient availability. Our findings demonstrated that the drought-tolerant citrus variety modulates its rhizosphere microbiome towards a more cooperative and resilient state, highlighting the critical role of host-specific microbial recruitment in enhancing plant adaptation to drought stress for sustainable agriculture.

## 1. Introduction

Water scarcity is a pervasive challenge for global agriculture, as drought stress resulting from it causes substantial economic losses worldwide annually [1]. In response to this intense natural selection pressure, plants have evolved intricate survival strategies over extended periods, encompassing multi-dimensional adaptive adjustments that range from macroscopic morphological changes to microscopic cellular modifications [2]. Enzymatic activities, i.e., peroxidase (POX), superoxide dismutase (SOD), and catalase (CAT) are significantly upregulated in plants during drought stress conditions [3,4,5]. Additionally, substances like proline within the osmotic adjustment system also exhibit elevated levels. This response effectively mitigates the accumulation of reactive oxygen species (ROS) and reduces water loss in leaf tissues, thereby enhancing the stress resistance efficiency in plants [6].

Recent studies have increasingly demonstrated that plants do not confront adverse conditions in isolation; rather, the microbial ecosystem surrounding their roots, the rhizosphere, plays an indispensable role as an ally in this process [7,8,9]. Rhizosphere microorganisms significantly influence the plant health and growth of their host plants by regulating soil nutrient transformation, promoting the secretion of plant growth substances, and decomposing organic residues. Notably, they exhibit considerable potential in mitigating environmental stresses, such as drought. A crucial insight emerging from recent studies is that the establishment of the rhizosphere microbiome is not a passive phenomenon; rather, it is likely actively shaped by the host plant’s genotype [10,11]. Distinct plant species, as well as different cultivars within a single species, have the capacity to select diverse microbial communities through variations in root architecture and significant differences in the composition of root exudates [12,13]. Drought-tolerant genotypes tend to selectively enrich specific microbial taxa that contribute to enhanced drought resilience capacity. These microorganisms include those capable of producing exopolysaccharides, which improve soil water-holding capacity (WHC), as well as those that synthesize plant growth-promoting hormones or antioxidants [14,15]. While numerous studies have documented the direct impacts of drought on soil microbial communities, there has been less extensive exploration into how drought sensitivity in host plants modulates these responses. Therefore, utilizing microbial resources as a sustainable ecological tool to enhance the environmental adaptability of crops is emerging as a cutting-edge focus in contemporary agricultural research. To achieve this objective, it is crucial to elucidate the response patterns and functional combination rules of rhizosphere microorganisms under drought conditions, thereby establishing a foundation for the effective utilization of this “living resource bank.”

As a cornerstone of the global horticultural industry, the yield and quality of citrus (*Citrus* spp.) have a direct impact on the livelihoods and nutrition of hundreds of millions of people globally [16]. The primary production areas are predominantly situated in tropical and subtropical regions that are susceptible to seasonal droughts. Insufficient water availability often results in a significant decline in yield productivity, with reductions reaching as high 50% in specific instances [17]. At the molecular level, transcriptomic and proteomic analyses have effectively identified drought-responsive genes and proteins involved in abscisic acid (ABA) signal transduction, photosynthesis, and stress resistance mechanisms [18,19,20,21]. These findings have further clarified the intrinsic genetic regulatory mechanisms that underlie citrus adaptation to stress conditions. In terms of nutrient uptake, drought conditions alter the availability of nutrients in the soil and the root system’s capacity for absorption [22]. Notably, this situation particularly inhibits the uptake of nitrogen, phosphorus, and potassium, thereby influencing the overall nutritional status and fruit quality of citrus trees [23]. The rhizosphere microbial community serves as a crucial interface between plant roots and the soil environment. Previous studies on fruit trees have primarily focused on how various covering measures reshape the structure of this microbial community [24,25]. However, when it comes to gaining a comprehensive understanding of the drought tolerance in citrus plants, research into rhizosphere microorganisms has remained a relatively overlooked aspect. Most related studies have focused on the mechanisms by which the inoculation of arbuscular mycorrhizal fungi (AMF) mitigates the adverse effects of drought stress on citrus plants [26,27]. However, there is a paucity of research examining how different genotypes of citrus influence the composition and structure of root-associated microorganisms. Therefore, incorporating the microbial dimension into the existing framework for understanding citrus drought tolerance is crucial for a comprehensive analysis of this phenomenon.

In the present study, we compared a citrus variety with poor drought tolerance (DS) and a variety with strong drought tolerance (DR) to test the central hypothesis that the drought tolerance of citrus varieties determines the structural and functional responses of their rhizosphere microbial communities to stress conditions. Our integrated analysis, which encompassed plant physiology, soil chemistry, enzyme activities, high-throughput sequencing, functional prediction, and microbial co-occurrence network analysis, aimed to evaluate rhizosphere differences between two varieties and reveal the impact of drought on microbial diversity and function. Furthermore, the investigation elucidated the varietal differences in microbial network stability under stress conditions. The findings of our study aim to establish the mechanistic interactions among plant genotypes, stress frequency, and the rhizosphere microbiota, offering novel insights for the development of microbiota-based strategies to improve stress tolerance efficiency.

## 2. Results

### 2.1. Variations in the Physicochemical Properties of Rhizosphere Soil and Physiological Indicators of Citrus Varieties Under Drought Stress

**Figure 1:**
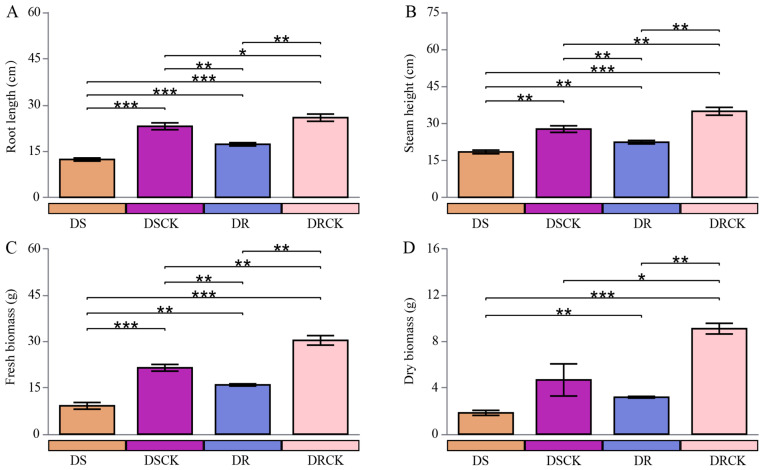
Variations in the stem height (**A**), root length (**B**), fresh (**C**), and dry weight (**D**) of different citrus varieties under drought stress conditions. The error bars indicate ± standard deviation, with n = 3 biological replicates. * indicates the significant levels at * *p* < 0.05, ** *p* < 0.01, and *** *p* < 0.001.

In the present study, a comparative analysis of growth metrics between two control groups, DSCK (drought-resistant varieties maintaining a soil moisture content of 65 to 55%) and DRCK (drought-resistant varieties maintaining a soil moisture content of 65 to 55%), was performed, revealing significant inherent disparities. Across all observed agronomic metrics, the values for the DRCK group consistently surpassed those of the DSCK group. Specifically, the average shoot height (34.9 ± 1.6 cm), root length (25.8 ± 1.2 cm), fresh weight (30.3 ± 1.5 g), and dry weight (9.1 ± 0.5 g) for the DRCK group were markedly better than those of the DSCK group, which had corresponding values of approximately 27.7 ± 1.3 cm, 23.1 ± 1.1 cm, 21.4 ± 1.1 g, and 4.7 ± 1.4 g, respectively. Compared to their respective control groups, both drought stress treatments (DS and DR) significantly inhibited plant growth and development. DS treatment resulted in a marked decline across all growth indicators when compared to the well-growing DSCK control group. The most pronounced reduction was observed in dry biomass (DB), which decreased from 4.7 to 1.8 g, indicating that drought stress severely impeded the accumulation of photosynthetic products in plants. Similarly, all growth indicators, i.e., shoot height (SH), root length (RL), fresh weight (FW), and dry biomass (DB) within the DR group were significantly lower than those in the DRCK control group. In the direct comparison of both stress treatments, the average shoot height (18.4 ± 0.7 cm), root length (12.3 ± 0.5 cm), fresh (9.2 ± 0.4 g), and dry weight (1.8 ± 0.1 g) were observed in the DS treatment significantly lower than the DR treatment (22.4 ± 0.8 cm, 17.2 ± 0.5 cm, 15.9 ± 1.1 g, and 3.2 ± 0.2 g) (Figure 1).

A distinct gradient relationship was evident among the four treatment groups across all growth indicators. The order of plant growth performance, from optimal to suboptimal conditions, was as follows: DRCK > DSCK > DR > DS. Under drought treatments, both citrus varieties exhibited significant growth inhibition; notably, the inhibitory effect induced by DS was markedly more pronounced than that of the DR group.

**Figure 2:**
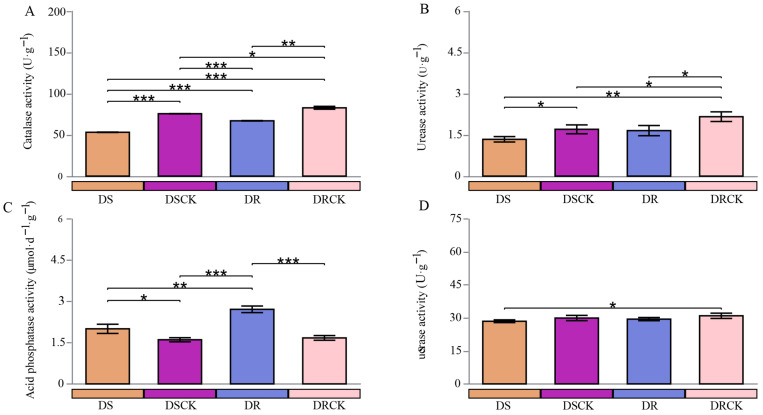
Differences in soil enzyme activities, such as catalase (**A**), urease (**B**), acid phosphatase (**C**), and sucrase (**D**) of different citrus varieties under drought stress conditions. The error bars indicate ± standard deviation, with n = 3 biological replicates. * represents the statistical significance levels at * *p* < 0.05, ** *p* < 0.01, and *** *p* < 0.001.

To gain an in-depth understanding of the effects of drought stress treatments on the rhizosphere microenvironments of various resistant varieties, this study assessed the activities of catalase (CAT), urease (UE), acid phosphatase (ACP), and invertase (SE) in the soil from each treatment group. The comparative analysis of soil enzyme activities in the DSCK and DRCK groups revealed that the activities of CAT, UE, and SE were significantly higher in the DRCK as compared to the DSCK group. This finding suggests that there are inherent differences in the soil biochemical environments between the two experimental systems under non-stress conditions. Conversely, the activities of acid phosphatase (ACP) had no significant differences (Figure 2).

In the context of stress effects, the DS and DR varieties exhibited distinct patterns in their impacts on various soil enzyme activities. The stress treatments consistently lowered CAT and UE activities. When compared to their respective control groups, DS and DR treatments resulted in a marked decline in CAT activity. Notably, the inhibitory effect of DS was particularly pronounced, with its CAT activity measured at 53.22 U·g^−1^, substantially lower than DR (67.15 U·g^−1^) (Figure 2A). Similarly, under the DS treatment, UE activity reached its lowest level. These findings indicated that both stresses reduced the soil antioxidant capacity and nitrogen transformation potential; however, the influence of the DS treatment was more profound (Figure 2B). Stress treatments resulted in a significant increase in acid phosphatase (ACP) activity. The ACP activity in DS and DR treatment groups was significantly higher than that observed in control groups. It is noteworthy that among these stress treatments, DR exhibited a markedly stronger enhancing effect on ACP activity, achieving the highest enzyme activity levels (Figure 2C). For sucrase (SE), differences among treatment groups were relatively low; no consistent or significant pattern of response to stress was discernible (Figure 2D).

**Figure 3:**
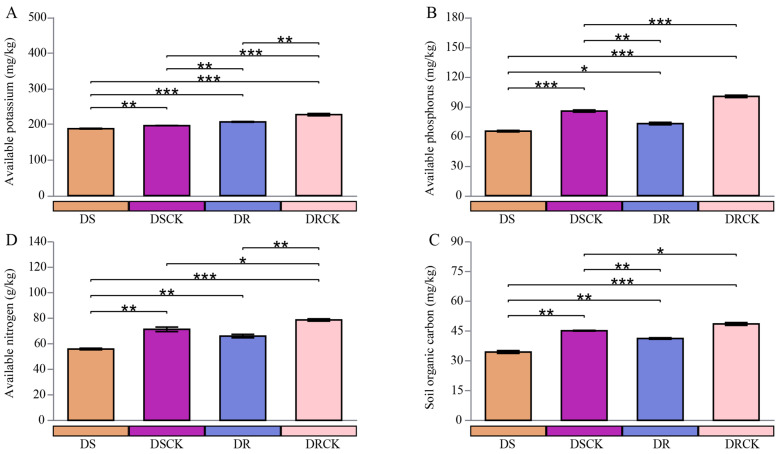
Characteristics of rhizosphere soil chemical properties, such as (**A**) available potassium (AK), (**B**) available phosphorus (AP), (**C**) available nitrogen (AN), and (**D**) soil organic carbon content (SOC) of citrus varieties with different stress tolerance efficiency under drought treatments. The error bars indicate ± standard deviation, with n = 3 biological replicates. * indicates the statistical significance levels at * *p* < 0.05, ** *p* < 0.01, and *** *p* < 0.001.

In the present study, both experimental treatments implemented (DS and DR) had significant negative impacts on key chemical properties of the soil, particularly with regard to nutrient availability and organic carbon storage (Figure 3). In comparison to the control group (DSCK), the DS treatment significantly reduced the levels of soil available potassium (AK), available phosphorus (AP), available nitrogen (AN), and soil organic carbon (SOC), respectively. The average SOC content in the DS treatment group was 34.4 g/kg, representing a 23.8% decrease compared to the DSCK group (45.1 g/kg) (Figure 3A). Furthermore, the soil microbial biomass carbon (MBC), microbial biomass phosphorus (MBP), and microbial biomass nitrogen (MBN) in the DS treatment group were measured at 240.3, 21.6, and 203.6 mg/kg, respectively. All of which were lower than observed in the DSCK group, which recorded values of 277.6 mg/kg for MBC, 30.6 mg/kg for MBP, and 215.7 mg/kg for MBN (Figure S1). Similarly, the contents of AK, AP, AN, SOC, MBC, MBP, and MBN in the DR treatment group were significantly lower than in the control group (DRCK). It is noteworthy that DS and DR resulted in significant reductions across all measured chemical indicators (AK, AP, AN, SOC) as well as microbial biomass indicators (MBC, MBN, MBP) relative to their respective control groups; there were distinct differences regarding the severity of impact on soil properties between these treatments. DS treatment resulted in a pronounced reduction across all indicators, with absolute values consistently lower than those of the other groups.

In contrast to this finding, although indicators within the DR treatment group were also significantly diminished when compared to those from DRCK, they exhibited a comparatively less severe degree of influence overall. Specifically concerning absolute values, the contents of AK, AP, AN, SOC, MBC, MBN, and MBP within the DR treatment cohort remained systematically higher than observed values under DS conditions. Moreover, when evaluating percentage (%) reductions relative to their respective control groups, it became evident that depletion resulting from DR treatment was notably less extensive than that induced by the DS intervention.

### 2.2. Diversity of Rhizosphere Microbial Communities of Different Citrus Under Drought Treatment

**Figure 4:**
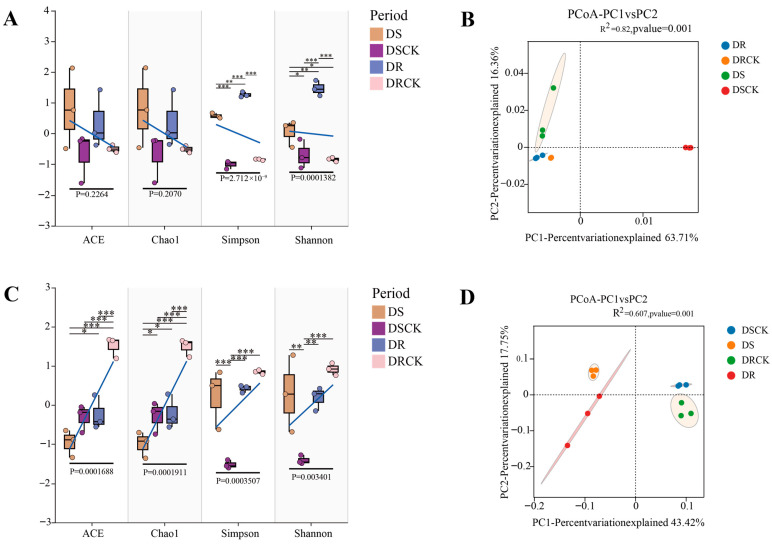
Analysis of diversity differences in bacteria and fungi in the rhizosphere soil of citrus under drought treatment (n = 3). The measured values of α diversity of rhizosphere bacteria and fungi in different treatments and varieties in terms of ACE, Chao 1 abundance, Shannon diversity, Simpson index (**A**,**C**), and principal coordinate analysis of bacteria and fungi diversity (**B**,**D**). α-diversity analysis was based on Student’s *t*-test and analyses of variance. The β-diversity distance algorithm employed the binary-Jaccard algorithm, and PERMANOVA was used for inter-group testing with a confidence level of 95%, indicating significant differences at * *p* < 0.05, ** *p* < 0.01, and *** *p* < 0.001.

To investigate the soil microbial structure of various plant varieties under drought conditions, we conducted α- and β-diversity analyses on the soil microorganisms across different treatment groups. The dilution curves of the samples tend to flatten as the sequencing data increase, indicating that the sequencing data are reasonable and can cover the bacterial (Figure S2) and fungal communities (Figure S3). The results showed that there were no significant differences in coverage among the various treatments, and the overall coverage was deemed satisfactory. At the bacterial level, analyses of the ACE and Chao1 index revealed no significant differences among the four treatment groups. This suggests that different treatments had a negligible impact on soil bacterial richness. However, when comparing the Shannon and Simpson indices, significant differences were observed across all treatment groups. Notably, substantial differences were found between DS and DR, as well as between drought treatments and control groups (DS vs. DSCK; DR vs. DRCK), with these differences being highly significant. This indicates that drought stress significantly influenced the evenness of the bacterial community, leading to considerable variations in the relative abundance of soil bacterial communities among different drought-resistant varieties under drought stress (Figure 4A,B). We conducted an Operational Taxonomic Units (OTUs) clustering analysis on all treatments utilizing principal coordinates analysis (PCoA) to investigate the differences in soil microbial composition between two varieties exhibiting distinct levels of drought tolerance under stress and control conditions. Our analyses revealed that bacterial and fungal communities could be categorized into four distinct clusters, effectively distinguishing between different treatments and varying drought-tolerant varieties. Further examination revealed that the bacterial community within the drought-treated and control groups was clearly separated into two distinct clusters. However, differentiation among various varieties under identical treatment conditions was not significant. In contrast, for fungi, the degree of community separation was more pronounced than observed in the bacterial community (Figure 4C,D).

**Figure 5:**
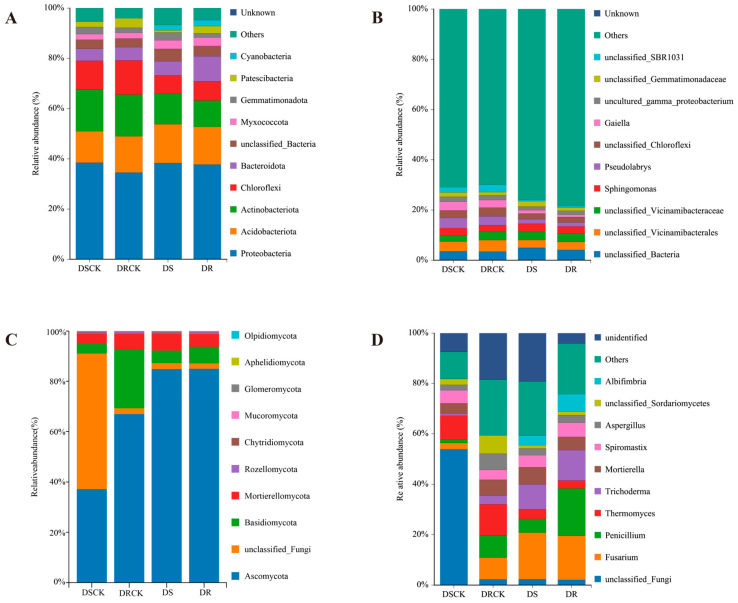
The relative abundance of bacterial and fungal communities in the rhizospheric soil of different citrus varieties in response to other treatments, i.e., CK and drought at the phylum level (**A**,**C**) and genus level (**B**,**D**).

In the bacterial community compositions at the phylum level across each treatment group, *Proteobacteria* emerged as the predominant phylum. Its relative abundance exceeded 34% in all treatment groups, with minimal differences observed among them. The other principal phyla included *Acidobacteriota*, *Actinobacteriota*, and *Chloroflexi*. The combined relative abundance of these three phyla surpassed 30%. Notably, when compared to the control groups (DSCK and DRCK), the relative abundances of *Actinobacteria* and *Chloroflexi* in the treatment groups of DS and DR exhibited a declining trend. The relative abundance of *Acidobacteriota* showed a slight increase in these treatment groups. Moreover, there was a remarkable increase in the proportion of *Bacteroidota* within the DR group (9.97%), which was approximately 1.5 to 2.0 fold higher than other groups. *Cyanobacteria* also demonstrated significant increases in the DS and DR groups (Figure 5A). At the genus level, the bacterial community was predominantly composed of a substantial number of low-abundance species, with the proportion of the “Others” category exceeding 69.9% in all instances. Among the major identifiable genera, the relative abundances of *Pseudolabrys* and *Gaiella* in the DS and DR treatment groups were significantly lower than those of the control groups (Figure 5B).

Analysis of the fungal community structure at the phylum level across different treatment groups revealed significant compositional differences. The phylum *Ascomycota* was predominant in the DS (84.87%) and DR (84.99%) groups, exhibiting a substantially higher proportion compared to the CK group. In contrast, unclassified fungi were notably abundant in the DSCK group (53.82%), but their prevalence significantly decreased in the DS, DRCK, and DR groups. While *Basidiomycota* demonstrated relatively high abundance in the DRCK group (23.39%), it declined to 6.63% in the DR group. However, this percentage remained significantly higher than that of the DS group, suggesting its potential role as a key taxon influencing drought resistance in citrus species (Figure 5C). In the analysis conducted at the fungal genus level, the genus *Fusarium* emerged as the most dominant in the drought-stressed and drought-resistant groups. Its abundance was significantly higher than that of the control group. Drought treatment notably increased the relative abundances of the genera *Penicillium* and *Trichoderma*. Conversely, a significant decrease in the abundance of *Thermomyces* was observed under drought conditions. Further analyses revealed that the DR treatment favored the enrichment of beneficial or functional fungi, including *Penicillium*, *Trichoderma*, and *Albifimbria*. Simultaneously, it significantly reduced the proportion of *unclassified taxa*. During drought conditions, DR treatment exerts more positive regulatory influence on fungal community structure compared to DS treatment (Figure 5D).

**Figure 6:**
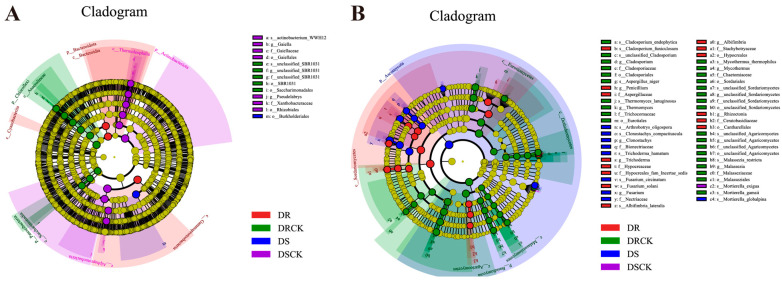
LEfSe analysis of each treatment group reveals the phylogenetic tree, which shows the distribution of bacterial (**A**) and fungal (**B**) lineages.

We utilized LEfSe to conduct a comprehensive analysis of rhizosphere microorganisms. The findings from the DSCK treatment revealed that the abundances of one phylum, two classes, two orders, and two families were significantly upregulated. In the DRCK treatment, there were notable increases in the abundances of two phyla, two classes, two orders, and one family (*unclassified_SBR1031*). For the DS treatment, an increase was observed solely in 1 order (*Burkholderiales*). In contrast, the DR treatment showed increased abundances for one phylum (*Bacteroidota*) and three classes (*Gammaproteobacteria*, *Bacteroidia*, *Cyanobacteria*) (Figure 6A). In addition, using an LDA score threshold of ≥4.0 as the criterion, we identified biomarkers of bacterial communities across different treatments (Figure 6B). Among the bacterial communities in DSCK, the top three were from the *f_Alphaproteobacteria*, *p_Actinobacteriota*, and *f_Thermoleophilia*. In DRCK, the predominant bacterial communities included *p_Chloroflexi*, *o_SBR1031*, and *o_Saccharimonadales*. In DS, only *o_Burkholderiales* within the bacterial community met this criterion. Conversely, in DR, the leading groups comprised *c_Gammaproteobacteria*, *c_Bacteroidia*, and *p_Bacteroidota* (Figure S4A).

The results of LEfSe analyses of fungi indicated that the number of enriched taxa in each treatment, except DSCK, increased significantly compared to bacteria. Specifically, DRCK exhibited enrichment in one phylum (*Basidiomycota*), four classes, five orders, six families, and six genera. DS showed enrichment in one phylum (*Ascomycota*), two families, and two genera. Meanwhile, DR demonstrated enrichment in one class (*Sordariomycetes*), two orders, five families, and four genera (Figure 6B). Based on the criterion of LDA ≥ 3.5, biomarkers of fungal groups under different soil carbon levels were determined. The top three biomarkers identified in the DRCK were *p_Basidiomycota*, *o_Eurotiales*, and *c_Eurotiomycetes*. In DSCK, only *s_Mortierella_exigua* met the criterion of having a score exceeding 3.5. In DS, the leading three biomarkers consisted of *p_Ascomycota*, *f_Nectriaceae*, and *g_Fusarium*. In DR, the primary biomarkers included *c_Sordariomycetes*, *o_Hypocreales*, and *g_Penicillium* (Figure S4B).

### 2.3. Functional Classification of Rhizosphere Microbial Communities

**Figure 7:**
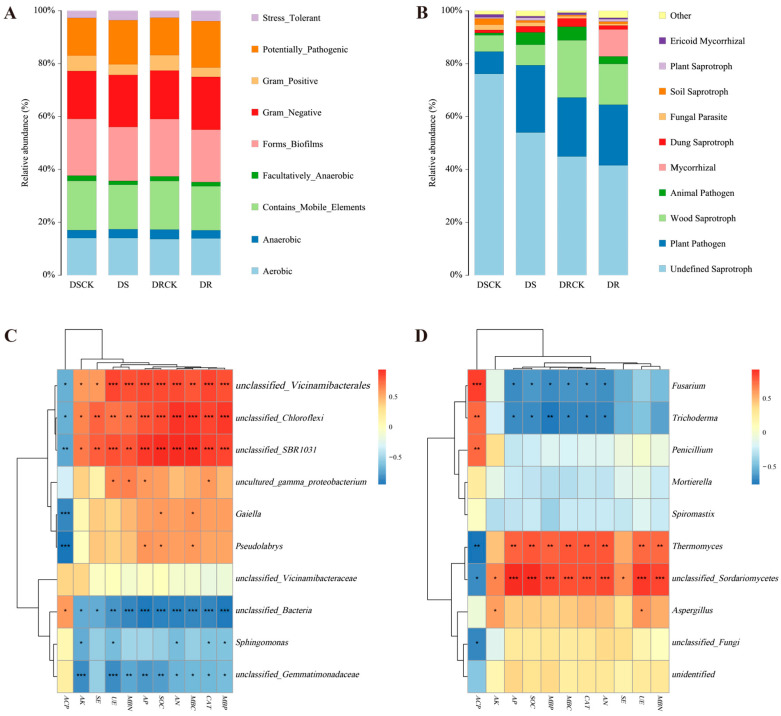
Functional classification of rhizospheric microorganisms in different citrus varieties and correlation analysis with soil properties. The bar charts present the classification of microbial functions for bacteria (**A**) and fungi (**B**). Panels (**C**) and (**D**) are heatmaps of the correlation analysis between soil physicochemical property indicators and the community structures of soil bacteria and fungi (at the genus level) based on Pearson analysis with a threshold of |r| ≥ 0.3 and *p* < 0.05. A red color indicates a positive correlation, while a blue color indicates a negative correlation. The deeper the color, the stronger the correlation. The symbols in the figures denote the significance levels at * *p* < 0.05, ** *p* < 0.01, and *** *p* < 0.001.

The phenotypic classification prediction of rhizosphere soil communities was carried out using BugBase (version 1.1.0). The results showed that the proportion of Gram-negative (Gram_Negative) bacteria in the treatment groups (DS-19.7% & DR-20.0%) was slightly higher than in the control groups (DSCK-18.1% & DRCK-18.3%). In contrast, the proportion of Gram-positive (Gram_Positive) bacteria showed a substantial decrease in the treatment groups. Specifically, the proportion in DS decreased by 31.7% compared to DSCK, and the proportion in DR decreased by 39.1% compared to DRCK. Forms_Biofilms and the Contains_Mobile_Elements exhibited lower abundances in the treatment groups, with the lowest values attained in the DR group. This implies that drought treatments (particularly in the DR treatment) may have suppressed specific population behaviors and genetic mobility of microorganisms. The stress-tolerant (Stress_Tolerant) phenotype was 3.5% in the DS treatment group and reached a maximum value of 4.0% in the DR treatment group. The values in both treatment groups were significantly higher than those in the DRCK control group (2.6%) and the DSCK control group (2.7%). Moreover, the potentially pathogenic (Potentially_Pathogenic) trait also exhibited an increasing trend in the treatment groups. This suggests that drought stress alters the rhizosphere environment, potentially selecting for microbial taxa that are better adapted to low moisture conditions and can utilize the specific root exudates released under stress conditions more efficiently (Figure 7A).

The fungal functional prediction results based on the FUNGuild database revealed that different treatments significantly modified the functional structure of the fungal community. As the most prevalent functional group, Undefined Saprotroph exhibited substantially lower relative abundance in the treatment groups (DS-54.0% & DR-41.5%) compared to the control groups (DSCK-76.2% & DRCK-44.9%). However, the functional abundance of plant pathogens increased substantially in the treatment groups. The abundances in the DS (25.4%) and DR (22.9%) groups were considerably higher than those in the corresponding control groups (DSCK, 8.4% & DRCK, 20.3%). Notably, Mycorrhizal fungi showed a remarkable enrichment in the DR group (10.2%), while their abundances were nearly negligible in the other groups (<0.1%). This represents one of the most unique functional features of the DR treatment (Figure 7B).

### 2.4. Correlation Analysis of Soil Characteristics and Microbial Community Structure and Co-Occurrence Network Analysis of Rhizosphere Microorganisms

The correlation analysis was performed between the top ten enriched microorganisms at the bacterial genus level and soil characteristics. The results revealed that the soil characteristic indicators, ACP and other indicators, could be distinctly classified into two groups, while the microorganisms were grouped into three categories. Specifically, *unclassified_Vicinamibacterales, unclassified_Chloroflexi, and unclassified_SBR1031* exhibited significant positive correlations with multiple environmental factors. In contrast, *Gaiella, Pseudolabrys, and unclassified_Gemmatimonadaceae* showed negative correlations with the majority of environmental factors. *Sphingomonas and unclassified_Vicinamibacteraceae* demonstrated a moderate degree of environmental responsiveness.

Meanwhile, *uncultured_gamma_proteobacterium and unclassified_Bacteria* indicated relatively weak environmental correlations (Figure 7C and Figure S5). There are obvious differences in the responses of different bacterial groups to environmental factors, and these differences may reflect the intrinsic driving forces behind changes in microbial community structure among various treatment groups. Among the fungi, *Fusarium* and *Trichoderma* were significantly negatively correlated with key environmental factors, whereas *Thermomyces* and *unclassified_Sordariomycetes* were significantly positively correlated with most environmental factors (Figure 7D and Figure S6). This result was highly consistent with the variation characteristics of the fungal community structure among the different treatment groups.

**Figure 8:**
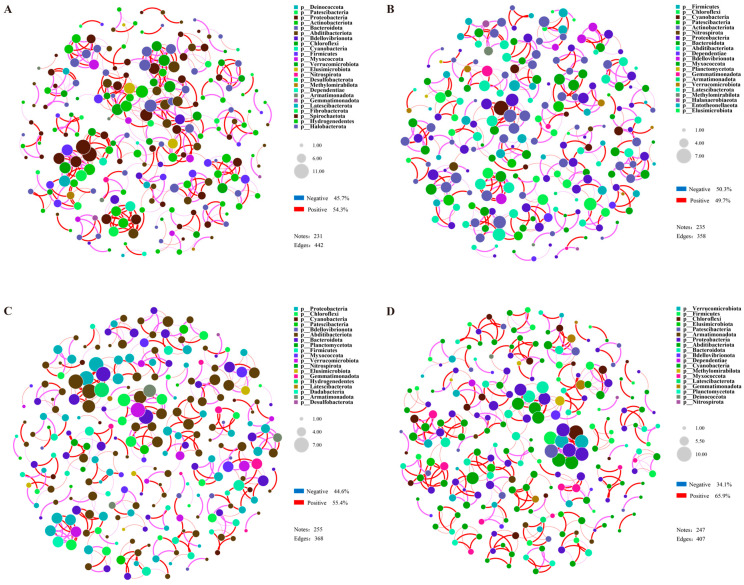
Network diagrams of rhizosphere bacteria under each treatment of DR (**A**), DRCK (**B**), DS (**C**), and DSCK (**D**). The size of the nodes in the figure represents species abundance, and different colors represent different species. The color of the connecting lines represents positive and negative correlations. Red lines indicate significant positive correlations, and pink lines indicate substantial negative correlations. The thickness of the lines represents the magnitude of the correlation coefficient, and Pearson correlation analysis was used. The default value of the correlation coefficient in the figure is *p* < 0.01. The number of lines indicates the closeness between nodes.

Co-linearity network analysis was conducted using the top 80 OTUs, aiming to explore the interactions among rhizosphere microorganisms of the drought-tolerant variety (DR) and the drought-sensitive variety (DS) under stress conditions. Overall, the number of nodes in the bacterial networks of the two treatments in DR was lower than in the two treatments of DS, while the opposite pattern was observed for fungi. Meanwhile, the number of edges and the density of correlation networks of bacteria in the two citrus varieties were significantly higher than those of fungi, indicating that the network structure of bacteria in the rhizosphere soil was more complex than that of fungi (Table S2). In the comparison between the drought treatment and the control group, the bacterial network of material DR under drought treatment was relatively more compact than that of DRCK, with the number of edges increasing by 23.5%. The proportion of positive correlations between bacterial OTUs rose from 49.7 to 54.3%, and the proportion of negative correlations decreased from 50.3 to 45.7% (Figure 8A,B). Similar to bacteria, the number of edges of fungi increased by 89.6%, and the number of nodes increased from 41 to 65. Still, its impact on the proportion of correlations among units was not significant (Figure S7A,B). In the two treatments of DS, the fungal networks were loose, with fewer edges and nodes compared to the DR treatment. Different from DR, under drought treatment, the bacterial network of DS was looser compared to DSCK. Despite having a greater number of nodes (DS-255 & DSCK-247), the number of edges decreased from 407 in DSCK to 368 in the drought-treated DS (Figure 8C,D). The proportion of positive correlations among bacterial OTUs decreased from 65.4 to 55.4%, and the proportion of negative correlations increased from 34.1 to 44.6% (Figure S7C,D). We used the network dissimilarity index to evaluate the differences in microbial networks. The results showed that there were two shared edges between DR and DS, with DR having 440 unique edges and DS having 366 unique edges. The network dissimilarity between the two reached 0.995 (Table S3). The results indicated that the complexity and variation patterns of the rhizosphere microbial networks of the two citrus materials under drought stress were significantly different, which might be closely associated with the differences in their drought resistance.

## 3. Discussion

### 3.1. Growth and Rhizosphere Microenvironment of Citrus with Different Genotypes

The seedling stage of plants serves as the foundation for their physiological development and is intricately linked to the formation of crop yields. A variety of abiotic stresses can undermine the normal growth of plants during the early growth phase, exerting persistent impacts and ultimately resulting in a reduction in the final crop yield [28]. Our study clearly demonstrates distinct differences in the physiological growth of drought-susceptible (DS) and drought-tolerant (DR) citrus genotypes under drought stress conditions. Specifically, DR exhibits significantly greater root length and shoot height compared to DS. In contrast, the biomass of DS is markedly reduced. Simultaneously, when comparing these genotypes with their respective well-watered control groups after the application of drought stress, plant growth status and substance accumulation showed significant reduction. This observation indicates that low water potential under drought conditions diminishes the nutrient absorption capacity of roots. Consequently, this leads to retarded growth and development in citrus seedlings.

Furthermore, the intrinsic relationship between substance synthesis and consumption within the plant becomes disrupted, resulting in the reduction in plant biomass [29]. DR is significantly less affected by drought factors than DS. Notably, the extent of decrease in the dry weight index is greater for DR (65%) than for DS (60.8%). This phenomenon may be attributed to more extensive root growth experienced by DR under drought stress, which necessitates higher energy consumption during its initial stages [30].

Our results revealed a more severe deterioration of rhizosphere conditions in DS compared to DR under drought, characterized by a coordinated decline in nutrients (AK, AP, AN, SOC), microbial biomass (MBC, MBN, MBP), and enzyme activities. This aligns with the known trend of drought-sensitive plants exacerbating rhizosphere stress [31]. The present results are strongly suggestive of an integrated syndrome linking the plant to its rhizosphere system [32]. Moreover, the observed superiority of the DR variety under control conditions (DRCK) underscores the potential role of genotype in pre-adapting the rhizosphere microenvironment for enhanced resilience [33].

### 3.2. Drought-Induced Reorganization of Root Microbial Communities in Citrus with Different Genotypes

The structural and functional adaptations of microbial communities to drought are reliant on soil type, tillage regime, and a variety of plant species or cultivars [34,35]. In this study, although drought stress did not significantly change the richness of bacteria, it substantially reshaped the structure and evenness of bacterial and fungal communities. The distinct separation in the principal coordinates analysis (PCoA) plots indicated that water deficit serves as a major driving force for microbial community assembly. Under drought stress, the fungal community showed higher sensitivity than the bacterial community (with more significant changes in the Chao1 index), which is contrary to the general belief that fungi have stronger drought tolerance but are more difficult to recover than bacteria [36,37]. This might be due to the fact that specific groups of ascomycetes can form symbiotic relationships with plants under short-term drought stress, and their relative abundance increases rapidly (Figure 5C). In previous studies, *Fusarium* was found to be more enriched as the severity of drought increased [38,39], a finding that is consistent with our results. In contrast, *Basidiomycota* significantly decreased with the intensification of drought. In wheat, its abundance was higher under moderate drought than under severe drought [40], which to some extent indicates that the DR rhizosphere environment was less affected by drought under the same treatment (the relative abundance of *Basidiomycota* was 34.6% higher in DR than in DS).

LEfSe analysis showed that the relative abundance of *Penicillium* and *Trichoderma* (*Basidiomycota*) increased significantly under drought conditions, suggesting that they may play a potential role in plants’ response to drought stress. These genera are well-documented biocontrol agents and plant growth promoters. *Trichoderma* species, for instance, have been associated with enhance plant drought tolerance by inducing systemic resistance and improving root growth [41,42,43]. Interestingly, mycorrhizal fungi were significantly and abundantly enriched only in the drought recovery treatment, and mycorrhizal fungi are widely recognized for their important role in enhancing plant drought resistance [44,45,46]. Although their functional significance in citrus requires further study, this indicates highly specific plant-fungal interactions. DR genotype was associated with enrichment of beneficial taxa under drought, potentially enhancing water and nutrient acquisition. This strategic recruitment of beneficial fungi by the DR variety contrasts with the passive microbial shifts in the DS variety, underscoring the host-genotype-dependent mediation of the stress response.

In terms of bacteria, the content of *Bacteroidetes* and *Patescibacteria* in the DR rhizosphere soil has increased. Different members of these phyla are fast-growing bacteria that thrive in nutrient-rich environments, such as the rhizosphere, and can promote nutrient cycling by collaborating with other species to enhance the drought resistance of crops [47,48,49]. This selective enrichment further suggests that the drought-tolerant plant genotype does not merely resist microbial change but actively shapes the functionally beneficial consortium under stress. However, the specific types of microbial communities recruited and the main effective species have only been predicted functionally. Future research needs to conduct further verification to directly confirm the colonization of these microorganisms on the root surface.

### 3.3. Functional Shifts in the Microbiome: From Pathogen Enrichment to Strategic Adaptation

The functional predictions reveal a complex picture of microbial strategy under drought. The increase in “Potentially Pathogenic” and “Stress Tolerant” bacterial phenotypes in treatments indicates the general shift towards a more competitive and resilient microbiome, a typical response to drought stress [50,51,52]. Under drought stress, the phenotypes of “forming biofilms” and “containing mobile genetic elements” were reduced, which is consistent with the findings of previous studies [53]. The fungal functional profile provides deeper insights. The universal decline in “Undefined Saprotrophs” and the surge in “Plant Pathogen” abundance align with the taxonomic rise of Fusarium. However, the DR variety was uniquely associated with a counterbalancing enrichment of “Mycorrhizal” fungi (Figure 7). This strategic functional shift suggests that the DR genotype fosters mutualistic relationships to enhance nutrient acquisition, thereby offsetting the negative impacts of the pathogen-enriched community. This functional redundancy or synergy between pathogenic and mutualistic guilds may be crucial for maintaining ecosystem functionality and plant fitness under stress conditions. The DR variety’s microbiome appears to be functionally more robust, capable of balancing competing demands, suppressing the negative effects of pathogens, while enhancing beneficial processes. However, as the prediction results of BugBase and FUNGuild are inferred from phylogenetic relationships, these functional predictions require further validation through metagenomics, transcriptomics, or pure culture experiments in future studies.

### 3.4. Correlation Analysis and Network Complexity Reveal the Mechanisms of Different Drought Resistances

The positive correlations between key bacterial groups, such as *Vicinamibacterales, Chloroflexi*, and *SBR1031* soil enzymes and nutrients highlight their indispensable role as integral components in the nutrient cycling mechanism [54,55,56,57], which is more tightly coupled within the DR rhizosphere. Co-occurrence network analysis provides a systems-level perspective on microbial interactions. The more complex, stable, and cooperative bacterial network in the DR variety under drought (characterized by increased edges and a higher proportion of positive correlations) is a hallmark of a resilient microbial community. In contrast, the simpler, more fragmented network in DS, with an increase in negative correlations, suggests a community under intense competitive pressure and ecological instability. This stark contrast implies that the drought-tolerant plant genotype fosters a more interconnected and synergistic root-associated microbiome. A robust microbial network likely enhances the functional stability of the rhizosphere, facilitating better nutrient cycling and stress protection, which collectively contribute to the superior drought tolerance of the DR citrus variety. The stability of the microbial network itself may be a critical factor, often overlooked in favor of taxonomic composition, which underpins the plant’s ability to withstand drought stress. As a correlative study, our work identifies strong associations but cannot definitively prove causation. Future experiments, such as reciprocal transplants of microbial communities or gnotobiotic studies with isolated keystone taxa, are needed to functionally validate the role of the identified microbiome in conferring drought tolerance.

## 4. Materials and Methods

### 4.1. Cultivar Selection and Field Experiment Design

This experiment was conducted in the greenhouse of Chongqing Three Gorges Vocational College, located in Wanzhou District, Chongqing, China, from April to July 2023. A drought-sensitive citrus material (GN-1, a bud mutation of Eureka 38, from Gan Ning in Chongqing) that was previously screened and selected, and drought-tolerant Wanzhou Red Tangerine plants collected from the Wanzhou Red Tangerine germplasm resource garden of Chongqing Three Gorges Vocational College were chosen as experimental materials and named as DS and DR, respectively, in this experiment. Sufficient seeds were obtained by collecting the fruits of the two citrus materials. After germination in an artificial climate chamber, the seeds are planted in the greenhouse’s planting pools. The same management measures were adopted for their cultivation. After 45 days, healthy seedlings of similar size and consistent growth conditions were selected and transplanted into flowerpots (diameter and height, 50 cm × 40 cm), keeping the fertile soil attached to their roots. The flowerpots were filled with the same amount of soil. A total of 40 pots were prepared, with 20 pots for each material. Each pot contained three citrus plants. After 5 days of acclimatization, 10 pots from each material were selected for subsequent drought treatment (DS and DR), while the remaining 20 pots were watered normally and named as DSCK and DRCK, respectively. Irrigation was carried out based on the weekly water requirement of citrus. During the initial planting period, the control plants were watered regularly and continuously to maintain soil moisture content (65 to 55%). For drought treatment, drought stress was induced by controlling water irrigation (soil moisture content at 35 to 25%), and the soil moisture content was measured using the TDR-100 soil moisture meter (Spectrum Technologies, Aurora, IL, USA).

### 4.2. Measurement of Soil Chemical Properties and Determination of Soil Enzyme Activities

At the end of drought stress (day 20), plants with consistent growth conditions were selected from each group. For each of the four treatments, rhizosphere soil from three independent pots (each pot considered one biological replicate) was collected. Soil from each pot was homogenized, subsampled, and analyzed, resulting in n = 3 biological replicates per treatment. The root systems were dug out along with the surrounding soil, and large soil clumps and stones were removed. Loose soil was gently shaken off, and the rhizosphere soil adhering to the root surface was collected with a brush. Subsequently, these samples were passed through a 2 mm sieve and thoroughly mixed. Each processed soil sample was then evenly divided into three parts, resulting in 12 independent soil samples (n = 12). Each soil sample was split into two parts, placed in self-sealing bags, and then transported in a sample box with ice packs at low temperature to the laboratory. One part was stored in a 4 °C refrigerator for subsequent analysis of soil enzyme activity, microbial biomass, carbon and nitrogen content, and available nutrient content. The other part was stored at −80 °C for microbial diversity sequencing.

Soil organic carbon (SOC) and soil available nitrogen (AN) were measured by the K_2_Cr_2_O_7_ wet oxidation and Kjeldahl methods [58]. The molybdenum-antimony-antimony colorimetric procedure was used to determine available phosphorus (AP). Available potassium (AK) was extracted with ammonium acetate and quantified by flame photometry. Microbial biomass nitrogen (MBN) was analyzed by the chloroform fumigation extraction method. Soil microbial biomass carbon (MBC) content was determined by the K_2_Cr_2_O_7_-H_2_SO_4_ external heating method. Soil microbial biomass phosphorus (MBP) was measured by the fumigation extraction method combined with colorimetry [59]. The potassium permanganate titration and sodium phenolate-sodium hypochlorite colorimetry were used to determine catalase (CAT) and urease (UE) activity. Alkaline phosphatase and sucrose were detected by sodium phenyl phosphate colorimetry and 3,5-dinitrosalicylic acid colorimetry [60]. All soil indicators and soil enzyme activities were measured with three replicates (n = 3) for each sample.

### 4.3. Soil Microbial DNA Extraction and High-Throughput Sequencing

Total genomic DNA was extracted from the soil samples using the TGuideS96 Magnetic Soil/Stool DNA Kit (Tiangen Biotech Co., Ltd., Beijing, China) according to the manufacturer’s instructions. The hypervariable region V3-V4 of the bacterial 16S rRNA gene was amplified using the primer pairs, specifically 338F (5′-ACTCCTACGGGAGGCAGCA-3′) and 806R (5′-GGACTACHVGGGTWTCTAAT-3′). The PCR products were assessed via agarose gel electrophoresis and subsequently purified utilizing the Omega DNA Purification Kit (Omega Inc., Norcross, GA, USA). The purified PCR products were collected, and paired-end sequencing (2 × 250 bp) was performed on the Illumina NovaSeq 6000 platform (Illumina Inc., San Diego, CA, USA). The Internal Transcribed Spacer (ITS) variable regions of fungi were amplified using primers ITS1FI2 (5′-GTGARTCATCGAATCTTTG-3′) and ITS2 (5′-TCCTCCGCTTATTGATATGC-3′). The ITS region, situated between the small subunit (SSU) and large subunit (LSU) ribosomal RNA genes, exhibits high variability and is commonly employed for fungal identification as well as phylogenetic analysis. The amplification procedure and conditions were identical to those used for the amplification of 16S rDNA. The resulting amplified products underwent high-throughput sequencing on the Illumina HiSeq 2500 platform, using a paired-end (PE) read length of 250 base pairs (bp). Sequencing services were provided by Beijing Biomarker Biotechnology Co., Ltd., Beijing, China.

### 4.4. Bioinformatic Analysis

The operational taxonomic units (OTUs) with a similarity threshold of 97% were clustered using UPARSE (version 10.0), and chimeric sequences were identified and removed [61]. The classification of each representative OTU sequence was analyzed against the 16S rRNA database (Silva v138) using the RDA Classifier (Randox Laboratories Ltd., Crumlin, UK) and compared against the ITS database through UNITE, with a confidence threshold of 0.7. α-diversity analysis was conducted using QIIME2 (V1.9.1) software to determine the species diversity and complexity of each sample [62]. β-diversity was observed using the Bray–Curtis distance algorithm and principal coordinate analysis (PCoA) to assess species complexity. One-way analysis of variance (ANOVA) was used to compare bacterial abundance and diversity. Linear discriminant analysis (LDA) combined with effect size (LEfSe) was applied to assess the differentially abundant taxa. The analysis was conducted based on the R software (version 4.1.3).

## 5. Conclusions

The current study elucidates that the drought sensitivity of various citrus cultivars significantly impacts the dynamics of their rhizosphere microbiome. The genotype exhibiting tolerance effectively modulates its associated microbial community, fostering a state characterized by enhanced cooperation, functional redundancy, and resilience. This transformation ultimately supports improved plant performance under conditions of water deficit. These findings underscore the vital role of host-mediated microbiome assembly in the adaptation of plants to stress, thereby providing a scientific foundation for the development of microbiome-based strategies. Such strategies may include the application of specific microbial consortia aimed at enhancing the efficiency of drought tolerance in crops.

## Data Availability

The raw data supporting the conclusions of this article will be made available by the authors on request.

## Supplementary Materials

The following supporting information can be downloaded at: https://www.mdpi.com/article/10.3390/plants15010077/s1. Figure S1.Characteristics of rhizosphere soil chemical properties of two citrus varieties with different drought tolerance under drought treatments. MBC: microbial biomass carbon, MBP: microbial biomass phosphorus, MBN: microbial biomass nitrogen. The meanings of the asterisks (*) in the figure are as follows: *, *p* < 0.05; **, *p* < 0.01; ***, *p* < 0.001. Figure S2. Analysis of dilution curves for bacterial sequencing. Among them, the x-axis represents the number of sampled sequences, and the y-axis represents the number of features. Figure S3. Analysis of dilution curves for fungus sequencing. Among them, the x-axis represents the number of sampled sequences, and the y-axis represents the number of features. Figure S4. A bar chart of biomarkers of bacterial (A) and fungal (B) taxonomic groups. Note: The red, blue and green dots represent significantly enriched bacteria and fungi respectively. In bacteria, the LDA value is ≥4, and in fungi, the LDA value is ≥3.5. Figure S5. The main active bacterial populations (at the genus level) influencing the community and environmental factors were analyzed through RDA (environmental factors are indicated by red arrows, and microorganisms by blue dashed arrows). Figure S6. The main active fungus populations (at the genus level) influencing the community and environmental factors were analyzed through RDA (environmental factors are indicated by red arrows, and microorganisms by blue dashed arrows). Figure S7. Network diagrams of rhizospheresoil fungi under each treatment (A) DR (B) DRCK (C) DS (D) DSCK. Note: The size of the nodes in the figure represents species abundance, and different colors represent different species. The color of the connecting lines represents positive and negative correlations: red lines indicate significant positive correlations, and blue lines indicate significant negative correlations. The thickness of the lines represents the magnitude of the correlation coefficient, and Pearson correlation analysis was used. The default value of the correlation coefficient in the figure is *p* < 0.01. The number of lines indicates the closeness between nodes. Table S1. Statistics of sample sequencing data processing results. Table S2. Key network metrics of bacterial communities in citrus varieties with different drought tolerance levels under various treatments. Table S3. Numbers of shared nodes and unique edges (correlations) and their dissimilarity between different rhizosphere bacteria sub-networks based on the different drought tolerance levels
