# Supplementary material for: Citrus Genotype Modulates Rhizosphere Microbiome Structure and Function Under Drought Stress
Source: Plants (Basel). 2025 Dec 26;15(1):77. doi: 10.3390/plants15010077 (PMC12787438; doi:10.3390/plants15010077)
Supplement: Supplementary file 1 [file plants-15-00077-s001.zip › plants-3976096-supplementary.pdf]

Supplementary Figures and Tables

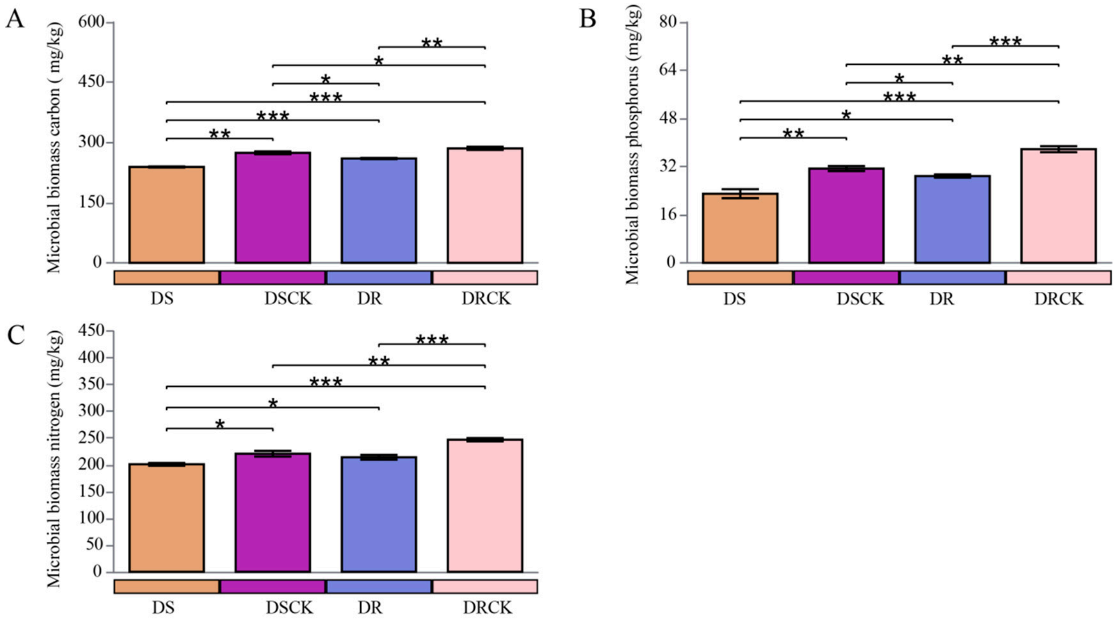

Figure S1. Characteristics of rhizosphere soil chemical properties of two citrus varieties with different drought tolerance under drought treatments. MBC: microbial biomass carbon, MBP: microbial biomass phosphorus, MBN:microbial biomass nitrogen. The meanings of the asterisks (*) in the figure are as follows: *, P < 0.05; **, P < 0.01; ***, P < 0.001.

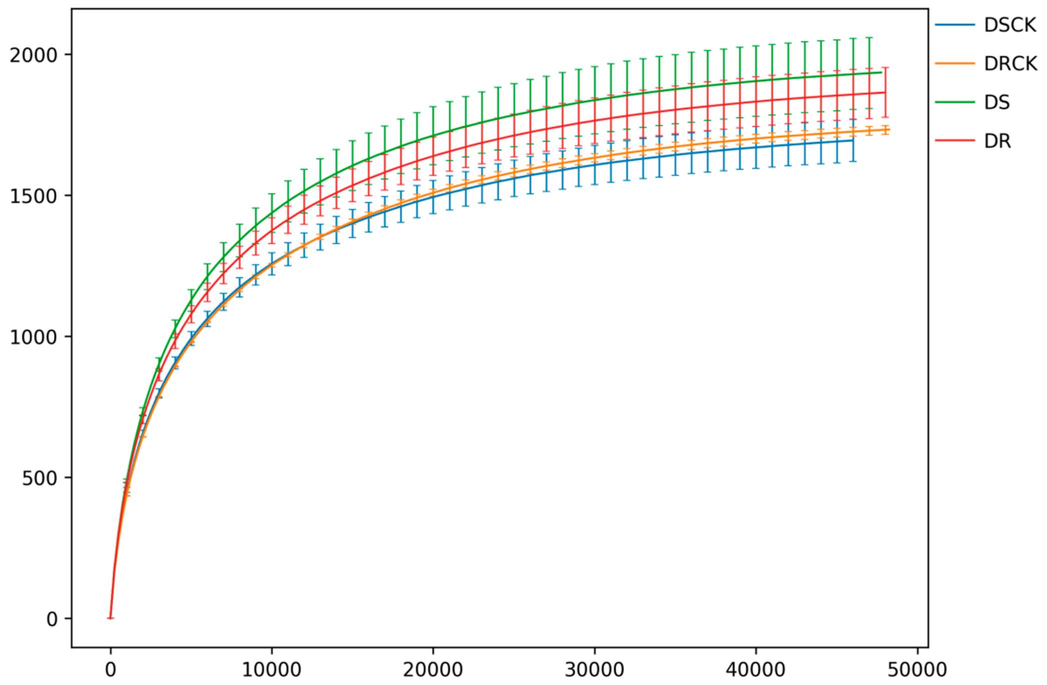

Figure S2. Analysis of dilution curves for bacterial sequencing. Among them, the x - axis represents the number of sampled sequences, and the y - axis represents the number of features.

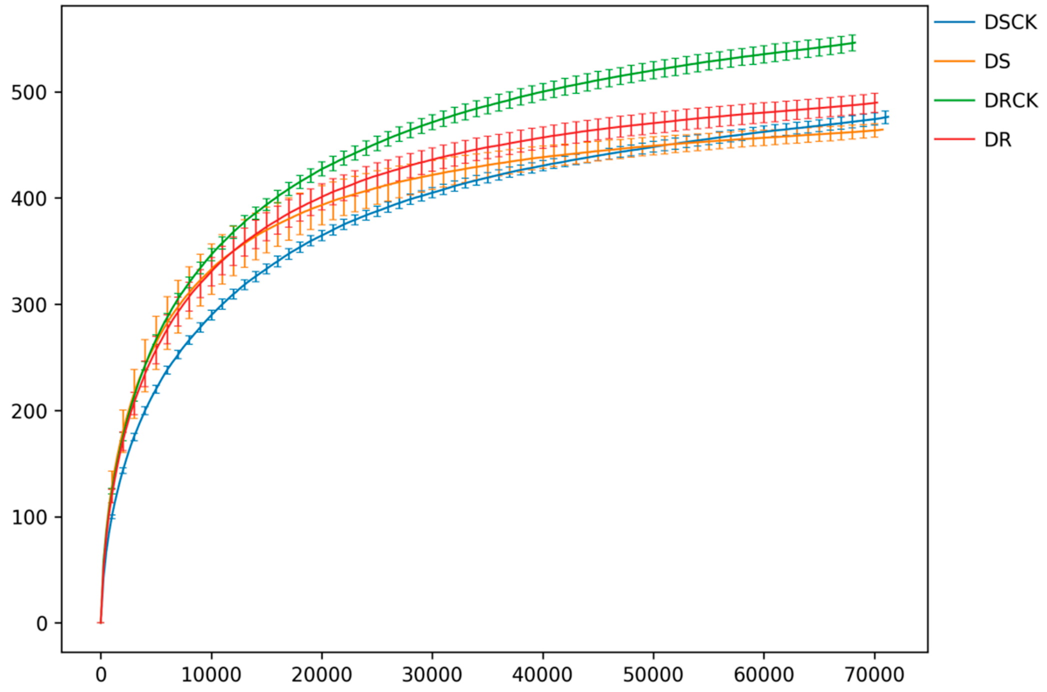

Figure S3. Analysis of dilution curves for fungus sequencing. Among them, the x - axis represents the number of sampled sequences, and the y - axis represents the number of features.

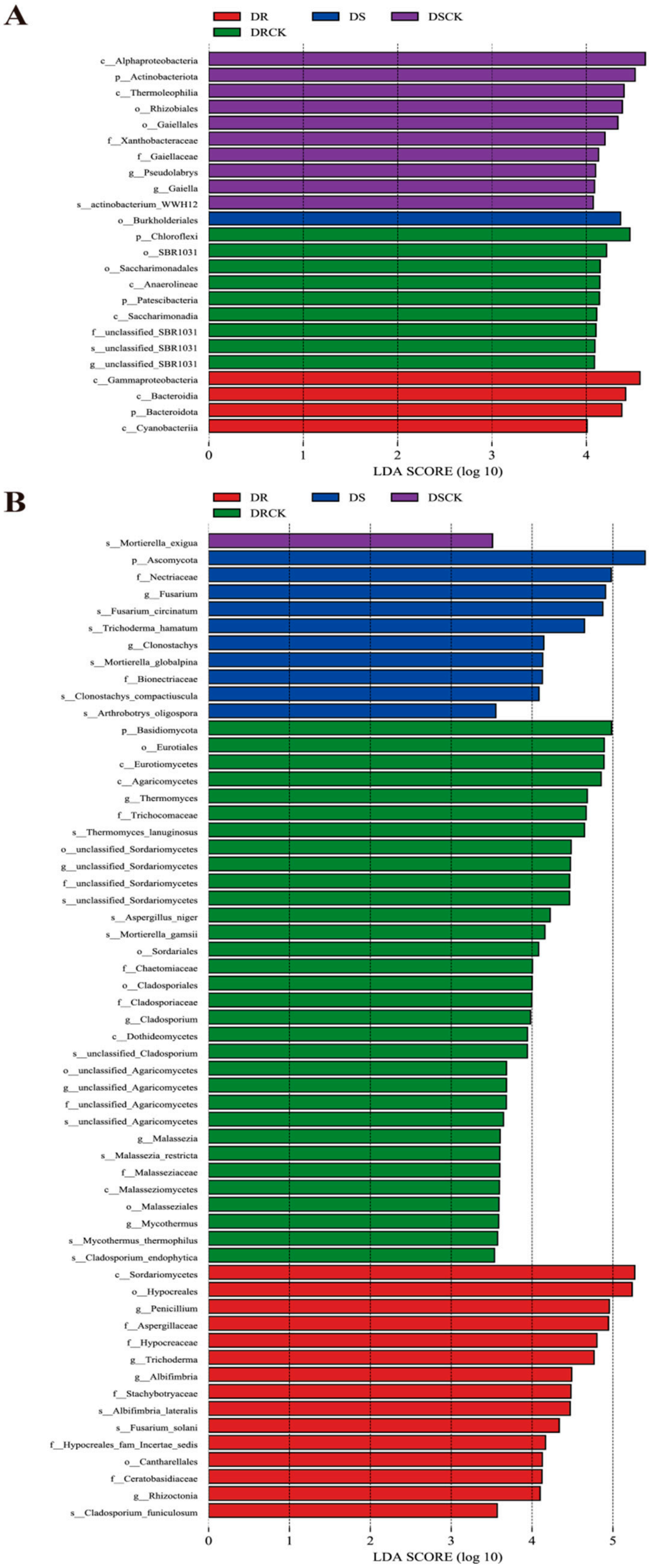

Figure S4. A bar chart of biomarkers of bacterial (A) and fungal (B) taxonomic groups. Note: The red, blue and green dots represent significantly enriched bacteria and fungi respectively. In bacteria, the LDA value is ≥ 4, and in fungi, the LDA value is ≥ 3.5.

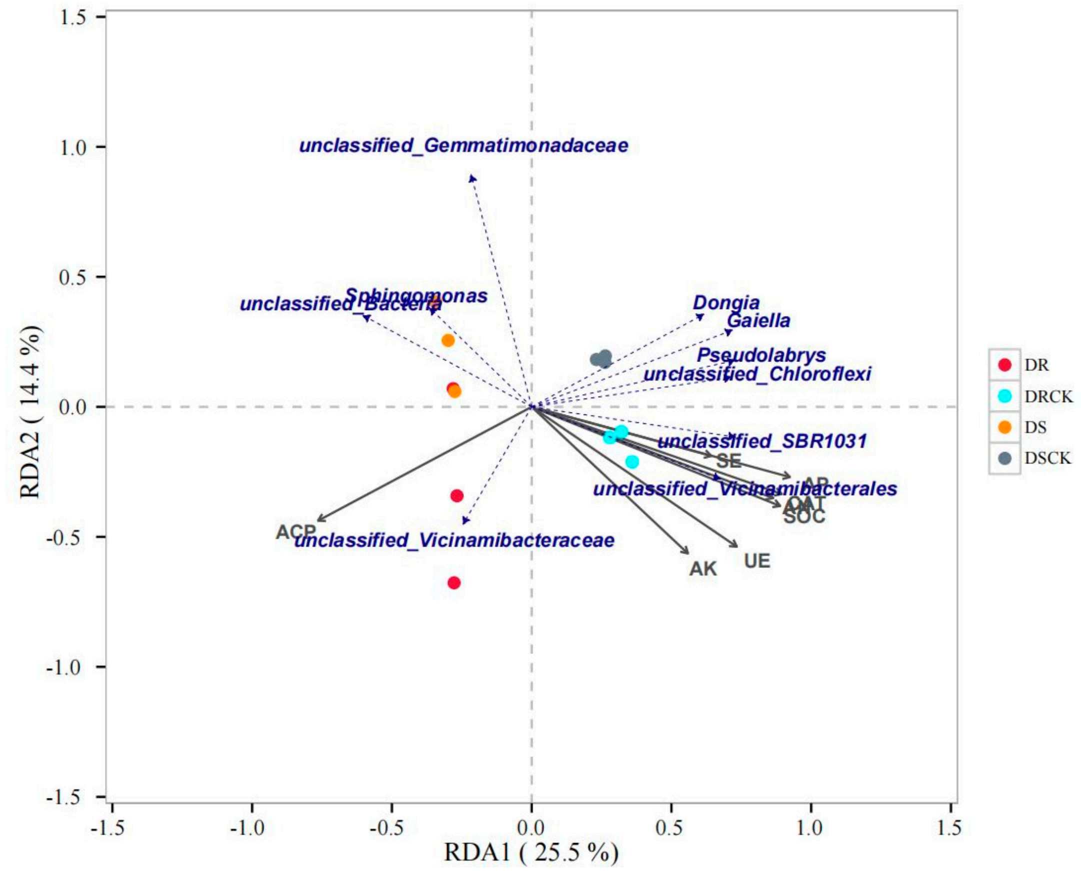

Figure S5. The main active bacterial populations (at the genus level) influencing the community and environmental factors were analyzed through RDA (environmental factors are indicated by red arrows, and microorganisms by blue dashed arrows).

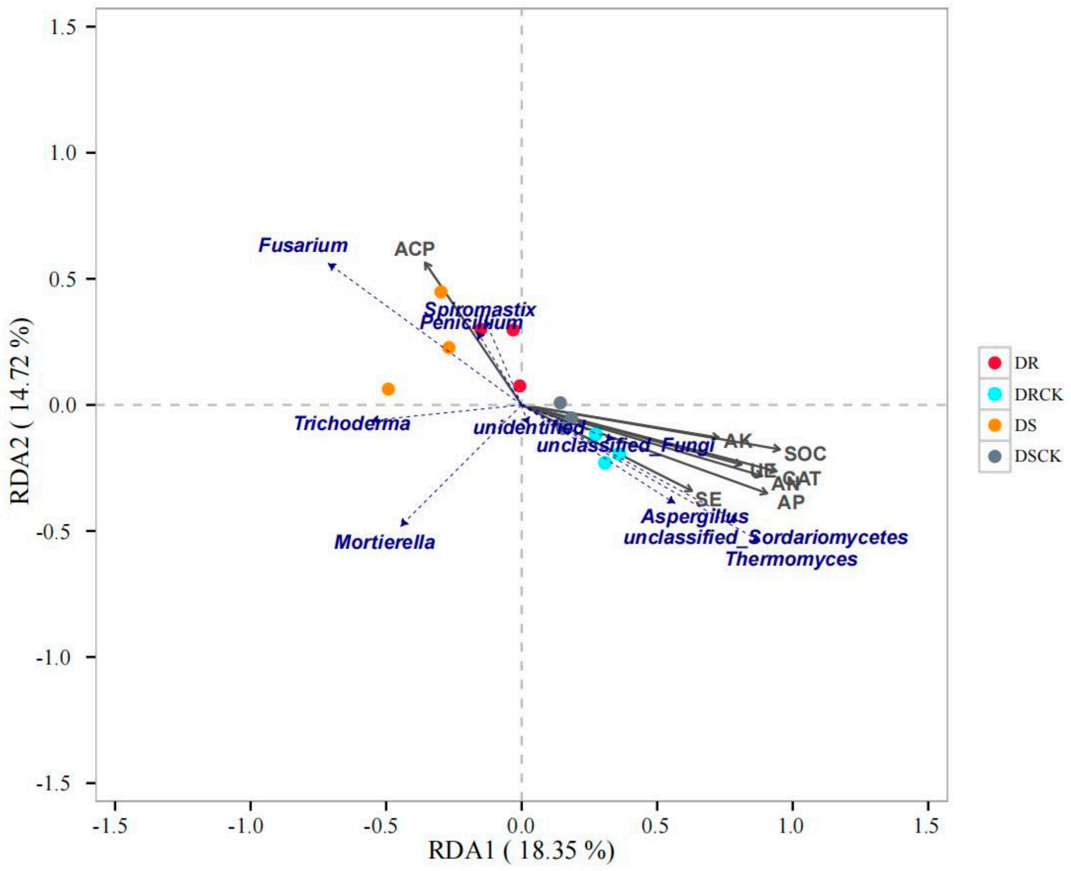

Figure S6. The main active fungus populations (at the genus level) influencing the community and environmental factors were analyzed through RDA (environmental factors are indicated by red arrows, and microorganisms by blue dashed arrows).

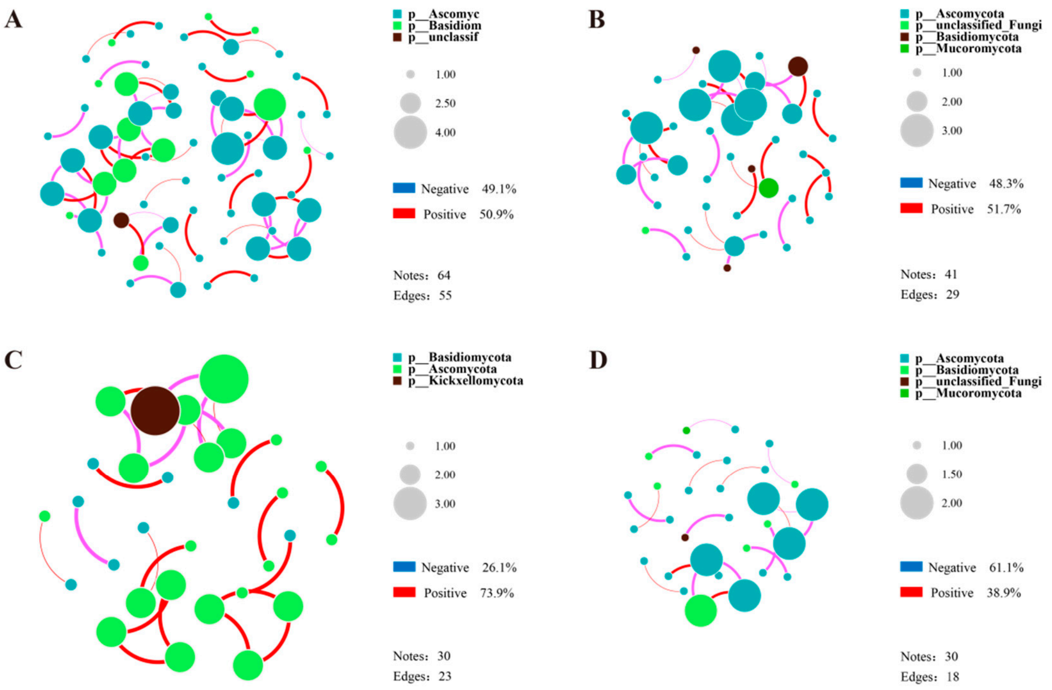

Figure S7. Network diagrams of rhizospheresoil fungi under each treatment (A) DR (B) DRCK (C) DS (D) DSCK. Note: The size of the nodes in the figure represents species abundance, and different colors represent different species. The color of the connecting lines represents positive and negative correlations: red lines indicate significant positive correlations, and blue lines indicate significant negative correlations. The thickness of the lines represents the magnitude of the correlation coefficient, and Pearson correlation analysis was used. The default value of the correlation coefficient in the figure is $p < 0.01$. The number of lines indicates the closeness between nodes.

Table S1. Statistics of sample sequencing data processing results

Sample ID	Raw Reads	Clean Reads	Denoised Reads	Merged Reads	Non-chimeric Reads
DSS1	79884	73859	72950	60478	55862
DSS2	79989	73406	72184	59305	53395
DSS3	79684	73587	72496	59733	54402
DSSCK1	79849	74286	73191	56973	51146
DSSCK2	79903	73277	72260	58147	53583
DSSCK3	80259	74624	73579	61539	57196
DSSMT1	80140	74592	73443	59834	54595
DSSMT2	76904	71169	69735	58189	51993
DSSMT3	79898	73369	72414	59824	54069
DTS1	79986	73507	72440	57768	52029
DTS2	80009	73222	72135	60419	54909
DTS3	80043	73147	72221	63108	57836
DTSCK1	80346	74968	73185	61691	56934
DTSCK2	80227	73609	71667	58707	53018
DTSCK3	79856	73757	72172	61244	56188
DTSMT1	80075	74749	73806	60079	54201
DTSMT2	75707	70022	68961	57453	51340
DTSMT3	80096	74002	72504	61154	55960

Table S2. Key network metrics of bacterial communities in citrus varieties with different drought tolerance levels under various treatments

Treatment	Number of Nodes	Number of Edges	Average Degree	Positive-to-Negative Edge Ratio
DR	231	442	3.83	1.19 (240:202)
DRCK	235	358	3.05	0.99 (178:180)
DS	255	368	2.89	1.24 (204:164)
DSCK	247	407	3.30	1.93 (268:139)

Table S3. Numbers of shared nodes and unique edges (correlations) and their dissimilarity between different rhizosphere bacteria sub-networks based on the different drought tolerance levels

Topological properties	Shared edges	Unique edges		Dissimilarity of networks
		DR	DS	
DR VS DS	2	440	360	0.995061728
